# Clinical and epidemiological characteristics of the dengue outbreak of 2024: a multicenter observation from Bangladesh

**DOI:** 10.1186/s41182-025-00691-y

**Published:** 2025-04-02

**Authors:** Mohammad Jahid Hasan, Muna Islam, Tanjina Tabassum, Md. Mohiuddin Khan, Md Khairul Islam, Rafiya Afroz, Rubaiya Rahman Tui, Mohammad Abdul Baset, Md. Anwar Syed, Joarder Rakeen Manzoor, Mohiuddin Sharif, Tamanna Tabassum

**Affiliations:** 1 Tropical Disease and Health Research Center, Dhaka, 1100 Bangladesh; 2 Department of Medicine, Chittagong Medical College Hospital, Chittagong, 4203 Bangladesh; 3 Pi Research and Development Center, Dhaka, 1100 Bangladesh; 4 Department of Medicine, Mymensingh Medical College Hospital, Charpara Medical Road, Mymensingh, 2200 Bangladesh; 5 Department of Medicine, Dhaka Medical College Hospital, Dhaka, 1100 Bangladesh; 6 Indoor Medical Officer, Department of Medicine, Shaheed Suhrawardy Medical College Hospital, Dhaka, 1207 Bangladesh

**Keywords:** Bangladesh, Dengue, Dengue hemorrhagic fever, Epidemiology, Flavivirus

## Abstract

**Background:**

Dengue fever remains a significant public health challenge in Bangladesh. This study aimed to characterize the clinical and epidemiological profiles of confirmed dengue cases during the 2024 outbreak in Bangladesh.

**Methods:**

This observational study was conducted from June to September 2024 in four tertiary care hospitals across Bangladesh located in four administrative divisions. A total of 401 laboratory-confirmed (NS1- or IgM-positive) dengue patients aged ≥ 12 years were included. Demographic data, clinical presentations, and laboratory findings were collected through face‒to-face interviews. The revised WHO 2009 dengue case classification was used for severity assessment of dengue patients. Descriptive and inferential statistics were used to summarize the results.

**Results:**

The mean age of patients with dengue fever was 29.81 ± 11.64 (SD) years, with 7.2% of the patients being adolescents (aged 12–17 years). A clear male predominance (88.3%) was observed. Overall, 65.6% of patients had dengue with warning signs, and 9% had severe dengue. Fever (94.3%), headache (70.3%), myalgia (66.1%), and gastrointestinal symptoms such as nausea (49.9%) and abdominal pain (43.9%) were common symptoms. The median in-hospital stay of both non-severe and severe cases were 4 & 5 days, respectively. The in-hospital mortality rate was 0.75%, which was significantly higher among severe dengue patients (5.6%).

**Conclusion:**

The 2024 dengue outbreak in Bangladesh predominantly affected young adult males, with a notable prevalence of gastrointestinal symptoms alongside classic dengue manifestations.

## Introduction

Dengue fever is a significant global health challenge with a history of continuous re-emergence over the past century [1]. This mosquito-borne viral infection is predominant in tropical and subtropical regions of the world affecting approximately 3.9 billion people residing in these areas [2], and each year, an estimated 400 million people are infected with the virus, leading to approximately 21,000 deaths [3]. Bangladesh, situated within a dengue-prone region, has experienced a complex history of dengue fever. The first case of dengue in Bangladesh was documented in the 1960s, followed by sporadic incidences until 1999 in Bangladesh, when the first major outbreak occurred in 2000, with 5551 hospitalizations and 93 deaths reported [4, 5]. Since then, the country has experienced recurrent dengue epidemics each year, with a notable increase in cases over time [6–8].

Dengue fever is caused by four serotypes of the dengue virus (DENV-1, DENV-2, DENV-3, and DENV-4), which present with a range of clinical features that vary from asymptomatic cases to severe dengue hemorrhagic fever and dengue shock syndrome [9]. The clinico-epidemiological landscape of dengue in Bangladesh has undergone significant transformations over the years [6]. The dominance of viral serotypes has shifted, with DENV-3 leading to numerous hospitalizations from 2000 to 2002 [10, 11] and DENV-2 dominating from 2013 to 2018, followed by a resurgence of DENV-3 from 2019 to 2022 [12, 13]. In 2023, DENV-2 once again emerged as the primary cause of cases and fatalities [14]. These serotype shifts have been accompanied by changes in clinical presentations. The early outbreaks in 2000 and 2002 were characterized by high-grade fever with typical purpuric rash and break-bone bodyache, whereas more recent outbreaks in 2010 and 2018 revealed a predominance of fever with gastrointestinal symptoms and bleeding manifestations, often with normal platelet counts [6, 15]. Similar to clinical presentations, the severity of dengue cases has also increased over time [13, 16]. Geographically, the disease has also expanded its reach, and recent outbreaks have shown a sharp increase in cases outside Dhaka, which is mostly the epicenter [17, 18].

The 2023 outbreak in Bangladesh was the deadliest worldwide that year, with over 321,000 hospitalizations and 1,705 deaths [19]. As of 2024, the country faces another outbreak of dengue infection, with a persistently increasing number of deaths and hospitalizations [20]. Until October, the reported number of cases was 41,810, with 210 deaths; however, the real number might be greater [21]. The changing trend of the clinico-epidemiological characteristics of dengue infection points to the need for continuous evaluation of each dengue outbreak in the local context of Bangladesh. Thus, this study aims to provide insights into the clinical profile of patients diagnosed with dengue fever during the 2024 outbreak in Bangladesh.

## Methods

### Study design and setting

This observational study was conducted from June 24 to September 24 during the dengue outbreak in Bangladesh. Four tertiary care government hospitals were selected across the country: Dhaka Medical College Hospital (DMCH) and Shaheed Suhrawardy Medical College Hospital (ShSMCH) in Dhaka, Mymensingh Medical College Hospital (MMCH) in Mymensingh, and Chittagong Medical College Hospital (CMCH) in Chittagong. The multicenter strategy was chosen to capture a comprehensive picture of the dengue outbreak of 2024 in Bangladesh.

### Study subjects

All patients aged 12 years and above, of both sexes, admitted to the studied hospitals (DMCH, ShSMCH, MMCH, and CMCH) with a confirmed diagnosis of dengue fever were considered for inclusion in this study. Dengue fever was confirmed on the basis of a positive result for either the dengue virus nonstructural protein 1 (NS1) antigen or the antidengue IgM antibody test. Any coinfection of dengue with other viral, protozoal, or bacterial infections was excluded from the study. Non-critically ill patients who were evaluated and sent home were not included in this analysis. Finally, a total of 401 patients were included as study patients.

### Study procedure

A semi-structured questionnaire was used to collect demographic information, clinical symptoms, and laboratory findings. Face‒to-face interviews were conducted by research physicians, and medical records were also reviewed thoroughly. A comprehensive clinical assessment was conducted on admission, including current symptoms; duration of symptoms; presence of gastrointestinal, respiratory, neurological, and bleeding manifestations; and evidence of plasma leakage, such as pleural effusion or ascites. In-hospital outcomes such as length of hospital stay, recovery status, and mortality (expired cases) were also recorded.

### Laboratory investigations

Blood samples were collected on admission for complete blood count, liver function tests (ALT, AST), and serological tests for dengue (NS1 antigen and IgM antibody). An automated hematology analyzer, Siemens ADVIA® 2120 (Munich, Germany: Siemens), was used to determine the complete blood count. For dengue virus detection, a dual diagnostic approach was employed. An InBios Dengue NS1 detection kit (InBios International, Inc., Seattle, WA, USA) was used for NS1 antigen detection. ELISA-based tests were performed to detect dengue-specific IgM antibodies. Liver function tests, including ALT and AST, were performed via a semiautomated biochemistry analyzer (Humalyzer 3000, Human Diagnostics, Germany) with commercially available reagent kits following standard operating procedures.

### Disease severity classification

Patients were classified according to the revised WHO 2009 dengue case classification as dengue without warning signs (group A), dengue with warning signs (group B) or severe dengue (group C) [22]. Group A consists of confirmed dengue cases without warning signs. Group B included patients with confirmed dengue who presented with any of the following warning signs: abdominal pain or tenderness, persistent vomiting, clinical fluid accumulation, mucosal bleeding, lethargy, restlessness, liver enlargement > 2 cm, or an increase in hematocrit (> 20%) concurrent with a rapid decrease in platelet count. These patients require in-hospital management and close observation. Group C, severe dengue, is characterized by severe plasma leakage, severe bleeding, severe organ impairment, or significant metabolic or electrolyte abnormalities. Disease severity was assessed on the basis of the clinical presentation on the day of admission.

### In-hospital outcomes

In addition to clinical assessments and laboratory investigations conducted on admission days, patient outcomes during hospitalization were evaluated, including the length of hospital stay, recovery status upon discharge and mortality rate (patients who died during hospitalization).

### Ethical considerations

The study was approved by the Ethical Review Committee of Dhaka Medical College (ERC-DMC/ECC/2024/135). Written informed consent was obtained from all adult participants (≥ 18 years) and from the legal guardians of the patients aged younger than 18 years.

### Statistical analysis

The data were analyzed via IBM SPSS Statistics, version 26.0. Descriptive statistics were used to summarize the demographic and clinical characteristics of the study population. Continuous variables are reported as the mean ± standard deviation (SD) and median (minimum–maximum), whereas categorical variables are presented as frequency and percentage. The clinical parameters were compared between adult and pediatric patients via appropriate statistical tests. The parameters were also compared between patients with nonsevere dengue and those with severe dengue along with in-hospital outcomes. Patients in Group A and Group B were considered to have nonsevere dengue, whereas those in Group C were considered to have severe dengue. For categorical variables, the Chi-square test with Yates’ continuity correction was applied. The normality of continuous variables was assessed using the Shapiro‒Wilk test, with a p < 0.05 indicating a nonnormal distribution. Normally distributed continuous variables were compared using an independent Student’s t test, whereas nonnormally distributed data were analyzed via the Mann‒Whitney U test. Univariate and multivariate logistic regression was also conducted. A p value of less than 0.05 was considered significant.

## Results

### Demographic characteristics

The study included a total of 401 patients with dengue fever, of which 7.2% (n = 29) were adolescents (aged 12–17 years), and the remaining 92.8% (n = 370) were adults. The mean age of the study population was 29.8 ± 11.6 (SD) years. The adolescents had a mean age of 15.93 ± 1.36 (SD) years, whereas the adults had a mean age of 30.12 ± 11.3 (SD) years. The majority of patients (60.3%) were in the 18–30 years age group.

Overall, 88.3% of the patients were male, and all the adolescents were male.

Most patients resided in urban areas (73.1%), whereas 25.1% lived in rural areas. The majority of patients (63.6%) reported a monthly family income between 20,000 and 40,000 Bangladeshi Taka (BDT). Comorbidities were observed in adults, with hypertension being the most common (10.3%), followed by diabetes mellitus (8.1%) (Table 1).

**Table 1:** Demographic characteristics of the patients with dengue fever (n = 401)

Characteristics	Total n = 401 n (%)	Adult n = 370 n (%)	Adolescents n = 29 n (%)
Age (years)			
12–17	29 (7.2)	–	29 (100)
18–30	242 (60.3)	242 (65.4)	–
31–40	61 (15.3)	61 (16.5)	–
41–50	37 (9.3)	37 (10)	–
51–60	22 (5.5)	22 (5.9)	–
> 60	8 (2)	242 (65.4)	–
Mean ± SD	29.8 ± 11.6	30.12 ± 11.3	15.93 ± 1.36
Gender			
Male	354 (88.3)	323 (87.3)	29 (100)
Female	47 (11.7)	47 (12.7)	0
Residence			
Urban	293 (73.1)	271 (74.2)	20 (69)
Rural	103 (25.1)	94 (25.8)	9 (31)
Monthly family income (BDT)			
< 20,000	99 (24.7)	89 (25.8)	9 (40.9)
20,000–40,000	255 (63.6)	242 (70.1)	13 (59.1)
40,001–60,000	8 (2)	8 (2.3)	0
> 60,000	6 (1.5)	6 (1.7)	0
Mean ± SD	23248.6 ± 13551	23529 ± 13844.63	19227.3 ± 6920.9
Comorbidities (Yes)			
Diabetes mellitus	30 (8.1)	30 (7.5)	0
Hypertension	41 (10.3)	41 (11.1)	0
COPD	4 (1)	4 (1.1)	0
CKD	1 (.3)	1 (.3)	0

*COPD* Chronic obstructive pulmonary disease, *CKD* Chronic kidney asthma

### Clinical presentations and laboratory findings

Fever was the most common presenting symptom, observed in 94.3% of patients, with a mean duration of 4.8 ± 2 days. Fever was significantly less common in adolescents (82.8%) than in adults (95.4%, p = 0.014). Other frequently reported general symptoms included headache (70.3%), myalgia (66.1%), and arthralgia (48.4%). Compared with adults, adolescents reported significantly fewer headaches (44.8%, p = 0.004) and myalgia (48.3%, p = 0.06).

Gastrointestinal symptoms were prominent, with nausea (49.9%), abdominal pain (43.9%), and anorexia (42.1%) being the most frequent presentations across all age groups. The neurological manifestations included dizziness in 15.7% of patients, with no significant differences between adolescents and adults.

Respiratory symptoms such as cough were observed in 24.9% of patients, whereas cardiovascular symptoms such as chest pain and palpitations were reported in 14.2% and 11.2%, respectively.

Bleeding manifestations occurred in some patients, with melena being the most common (14%), followed by gum bleeding (6%) and hematemesis (5%). Signs of plasma leakage were noted in 36.7% of patients (Table 2).

**Table 2:** Comparison of clinical presentations between adult and adolescent dengue patients (n = 401)

Clinical presentations	Total n = 401 n (%)	Adult n = 370 n (%)	Adolescent n = 29 n (%)	p value
General symptoms				
Fever	378 (94.3)	353 (95.4)	24 (82.8)	0.014*
Duration of fever (days) (Mean ± SD)	4.8 ± 2	4.7 ± 2.02	4.60 ± 1.5	0.19*
Headache	282 (70.3)	267 (72.2)	13 (44.8)	0.004*
Myalgia/muscle pain	265 (66.1)	249 (67.3)	14 (48.3)	0.06*
Arthralgia/bone pain	194 (48.4)	182 (49.2)	10 (34.5)	0.18*
Fatigue/lethargy	23 (5.7)	23 (6.2)	0	0.33*
Restlessness	22 (5.5)	10 (2.7)	0	0.35*
Rash	20 (5)	19 (5.1)	1 (3.4)	1*
Dehydration	28 (7)	27 (7.3)	1 (3.4)	0.68*
Neurological manifestations				
Dizziness	63 (15.7)	60 (16.2)	3 (10.3)	0.56*
Vertigo	15 (3.7)	14 (3.8)	1 (3.4)	1*
Hallucinations	12 (3)	12 (3.2)	0	0.67*
Confusion	10 (2.5)	10 (2.7)	0	0.78*
Loss of consciousness	10 (2.5)	10 (2.7)	0	0.78*
Convulsion	2 (0.5)	2 (0.5)	0	1*
Gastrointestinal manifestations				
Nausea	200 (49.9)	188 (50.8)	10 (34.5)	0.13*
Anorexia	169 (42.1)	159 (43)	8 (27.6)	0.15*
Abdominal pain	176 (43.9)	160 (43.2)	15 (51.7)	0.15*
Acute vomiting	138 (34.4)	129 (34.9)	8 (27.6)	0.54*
Persistent vomiting (> 3 times/day)	67 (16.7)	64 (17.3)	2 (6.9)	0.23*
Persistent diarrhea (> 3times/day)	96 (23.9)	89 (24.1)	6 (20.7)	0.85*
Abdominal distension	23 (5.7)	21 (5.7)	1 (3.4)	0.93*
Hepatomegaly	8 (2)	8 (2.2)	0	0.91*
Splenomegaly	1 (0.2)	1 (0.3)	0	1*
Ascites	48 (12)	46 (12.4)	1 (3.4)	0.25*
Respiratory manifestations				
Cough	100 (24.9)	93 (25.1)	5 (17.2)	0.46*
Respiratory distress	25 (6.2)	24 (6.5)	0	0.31*
Breathlessness	4 (1)	4 (1.1)	0	0.57*
Hemoptysis	9 (2.2)	8 (2.2)	0	0.91*
Cardiovascular manifestations				
Chest pain	57 (14.2)	55 (14.9)	1 (3.4)	0.15*
Palpitation	45 (11.2)	40 (10.8)	4 (13.8)	0.85*
Shock	20 (5)	19 (5.1)	1 (3.4)	1*
Pale, cold, clammy hands and feet	21 (5.2)	21 (5.7)	0	0.37*
Cyanosis	1 (0.2)	1 (0.3)	0	1*
Bleeding manifestations				
Melena	56 (14)	52 (14.1)	4 (13.8)	1*
Hematemesis	20 (5)	19 (5.1)	1 (3.4)	1*
Gum bleeding	24 (6)	21 (5.7)	2 (10.3)	0.54*
Epistaxis	16 (4)	16 (4.3)	0	0.51*
Hematuria	3 (0.7)	3 (0.8)	0	1*
Excessive menstrual bleeding	1 (0.2)	1 (0.3)	0	1*
Ocular manifestations				
Red eye	38 (9.5)	32 (8.6)	6 (20.7)	0.072*
Features of plasma leakage	147 (36.7)	133 (35.9)	13 (44.8)	0.45*
Hematological laboratory findings (Median)				
HCT (%)	40.8 (24.2–56.6)	40.7 (24.2–56.6)	41.2 (26.1–51.5)	0.99
Hb (gm/dl)	13.35 (7–19.5)	13.3 (7–19.5)	13.4 (7.9–16.2)	0.86
WBC (cells/μl)	5200 (1200–15300)	5200 (1640–15300)	5550 (1200–11000)	0.91
Platelet (cells/μl)	83000 (10000–374000)	76000 (15000–257000)	84500 (10000–374000)	0.06
Serum levels of liver enzymes (Mean ± SD)				
ALT (U/L)	76.50 (15–670)	78 (15–461)	57.5 (15–272)	0.19
AST (U/L)	87.5 (14–538)	88 (14–538)	89.5 (23–538)	0.43

The data are presented as n (%) unless otherwise specified

*HCT* hematocrit, *Hb* hemoglobin, *WBC* white blood cell count, *ALT* alanine aminotransferase, *AST* aspartate aminotransferase

The p value was determined by the chi-square test with Yates’ continuity correction*, independent Student’s t test** and the Mann‒Whitney U test

The median hematocrit level was 40.8% (range: 24.2–56.6%), the hemoglobin level was 13.35 g/dL (range: 7–19.5), the white blood cell count was 5,200 cells/μL (range: 1,200–15,300), and the platelet count was 83,000 cells/μL (range: 10,000–374,000). The median ALT and AST levels were 76.5 U/L (range: 15–670) and 87.5 (range: 14–538), respectively (Table 2).

### The severity of dengue

**Fig. 1:**
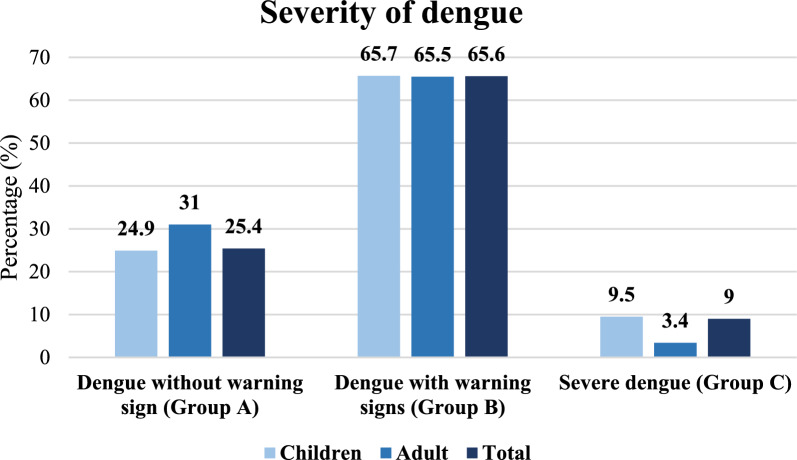
Severity of dengue cases according to the revised WHO dengue case classification (n = 401)

On the basis of the revised WHO dengue case classification, the severity distribution among the studied patients was assessed, with 25.4% of patients in Group A (dengue without warning signs), 65.6% in Group B (dengue with warning signs), and 9% in Group C (severe dengue). No significant difference was observed in the severity of disease between children and adults (Fig. 1).

The age and sex distributions were statistically similar between patients with nonsevere dengue and those with severe dengue. Patients with severe dengue presented these symptoms more frequently, including myalgia, fatigue, dizziness, abdominal distension, ascites, cough, breathlessness, shock, and features of plasma leakage. Hematocrit levels were significantly greater in patients with severe dengue, whereas platelet counts were significantly lower. White blood cell counts and hemoglobin levels were statistically similar between the two groups. ALT and AST levels were statistically similar between the two groups (Table 3).

**Table 3:** Clinical presentation of severe and nonsevere dengue patients (n = 401)

Variable	Nonsevere dengue n = 364 n (%)	Severe dengue n = 36 n (%)	p value
Age	29.66 ± 11.7	31.41 ± 11.4	0.38**
Gender			
Male	322 (88.2)	32 (88.9)	0.90*
Female	43 (11.8)	4 (11.1)	
General symptoms			
Fever	346 (94.8)	32 (88.9)	0.14*
Duration of fever (days) (Mean ± SD)	4.69 ± 1.9	4.88 ± 2.4	0.69**
Headache	253 (69.3)	29 (80.6)	0.15*
Myalgia/muscle pain	233 (63.8)	32 (88.9)	0.002*
Arthralgia/bone pain	175 (47.9)	19 (52.8)	0.58*
Fatigue/lethargy	13 (3.6)	10 (27.8)	< 0.001*
Restlessness	19 (5.2)	3 (8.3)	0.43*
Rash	16 (4.4)	4 (11.1)	0.07*
Dehydration	22 (6)	6 (16.7)	0.02*
Neurological manifestations			
Dizziness	46 (12.6)	17 (47.2)	< 0.001*
Vertigo	11 (3)	4 (11.1)	0.01*
Hallucinations	12 (3.3)	0	0.26*
Confusion	9 (2.5)	1 (2.8)	0.90*
Loss of consciousness	8 (2.2)	2 (5.6)	0.22*
Convulsion	1 (.3)	1 (2.8)	0.04*
Gastrointestinal manifestations			
Nausea	183 (50.1)	17 (47.2)	0.73*
Anorexia	152 (41.6)	17 (47.2)	0.51*
Abdominal pain	156 (42.7)	20 (55.6)	0.13*
Acute vomiting	125 (34.2)	13 (36.1)	0.82*
Persistent vomiting (> 3 times/day)	58 (15.9)	9 (25)	0.16*
Persistent diarrhea (> 3times/day)	83 (22.7)	13 (36.1)	0.07*
Abdominal distension	18 (4.9)	5 (13.9)	0.02*
Hepatomegaly	6 (1.6)	2 (5.6)	0.109*
Splenomegaly	0	1 (2.8)	0.001*
Ascites	39 (10.7)	9 (25)	0.012*
Respiratory manifestations			
Cough	86 (23.6)	14 (38.9)	0.04*
Respiratory distress	21 (5.8)	4 (11.1)	0.21*
Breathlessness	2 (0.5)	2 (5.6)	0.004*
Hemoptysis	7 (1.9)	2 (5.6)	0.16*
Cardiovascular manifestations			
Chest pain	49 (13.4)	8 (22.2)	0.14*
Palpitation	40 (11)	5 (13.9)	0.59*
Shock	0	20 (55.6)	< 0.001*
Pale, cold, clammy hands and feet	6 (1.6)	15 (41.7)	< 0.001*
Bleeding manifestations			
Melena	50 (13.7)	6 (16.7)	0.62*
Hematemesis	17 (4.7)	3 (8.3)	0.34*
Gum bleeding	20 (5.5)	4 (11.1)	0.17*
Epistaxis	13 (3.6)	3 (8.3)	0.16*
Hematuria	2 (.5)	1 (2.8)	0.13*
Excessive menstrual bleeding	1 (.3)	0	0.75*
Ocular manifestations			
Red eye	32 (8.8)	6 (16.7)	0.12*
Features of plasma leakage	114 (31.2)	33 (91.7)	< .001*
Hematological laboratory findings Median (Minimum–Maximum)			
HCT (%)	40.5 (24.7–56.6)	44.7 (24.2–55)	0.014〹
Hb (gm/dl)	13.3 (7–19.5)	13.8 (8.2–18)	0.39〹
WBC (cells/μl)	5200 (1370–15300)	5215 (1200–11380)	0.83〹
Platelet (cells/μl)	88500 (10000–374000)	52500 (14000–328000)	0.003〹
Serum levels of liver enzymes (Mean ± SD)			
ALT (U/L)	76 (15–461)	78 (16–232)	0.94〹
AST (U/L)	90.7 (14–538)	57 (22–129)	0.17〹

The data are presented as n (%) unless otherwise specified

*HCT* hematocrit, *Hb* hemoglobin, *WBC* white blood cell count, *ALT* alanine aminotransferase, *AST* aspartate aminotransferase

The p value was determined via the chi-square test*, independent Student’s t test** and the Mann‒Whitney U test〹

Univariate regression analysis identified several predictors of severe dengue, including fatigue/lethargy, pale, cold, clammy extremities, dizziness, features of plasma leakage, myalgia/muscle pain, dehydration, abdominal distension, vertigo, and breathlessness. In the multivariate analysis, fatigue/lethargy, pale, cold, clammy extremities, features of plasma leakage, and dizziness were independently associated with severe dengue (Table 4).

**Table 4:** Predictors of severe dengue (n = 401)

Clinical presentations	Univariate OR (95% CI)	p value*	Multivariate OR (95% CI)	p value**
Age	1.01 (0.98–1.04)	0.38	–	
Myalgia/muscle pain	4.53 (1.5–13.09)	0.005	3.17 (0.99–10.14)	0.05
Fatigue/lethargy	10.41 (4.16–26.01)	< 0.001	9.54 (3.07–29.60)	< 0.001
Dehydration	3.11 (1.17–8.28)	0.02	2.08 (0.48–9.01)	0.32
Dizziness	6.21 (3.1–12.79)	< 0.001	2.55 (1.03–6.26)	0.04
Vertigo	4.02 (1.21–13.35)	0.02	1.61 (0.34–7.59)	0.54
Convulsion	0.09 (0.01–1.57)	0.1	–	
Abdominal distension	3.10 (1.08–8.94)	0.04	3.12 (0.94–10.36)	0.06
Ascites	2.78 (1.22–6.35)	0.02	1.67 (0.51–5.45)	0.54
Cough	2.06 (1.01–4.20)	0.04	1.34 (0.57–3.14)	0.49
Breathlessness	10.67 (1.45–78.20)	0.02	9.95 (0.98–100.3)	0.05
Pale, cold, clammy hands and feet	42.73 (15.04–121.40)	< 0.001	18.40 (6.70–55.70)	< 0.001
Features of plasma leakage	24.21 (7.27–80.68)	< 0.001	21.71 (4.96–94.96)	< 0.001
Hematocrit	1.06 (1.003–1.127)	0.04	1.05 (0.97–1.17)	0.178

*AST* aspartate aminotransferase, *OR* Odds ratio

The p value was determined by univariate* and multivariate logistic regression**

### In-hospital outcomes

**Fig. 2:**
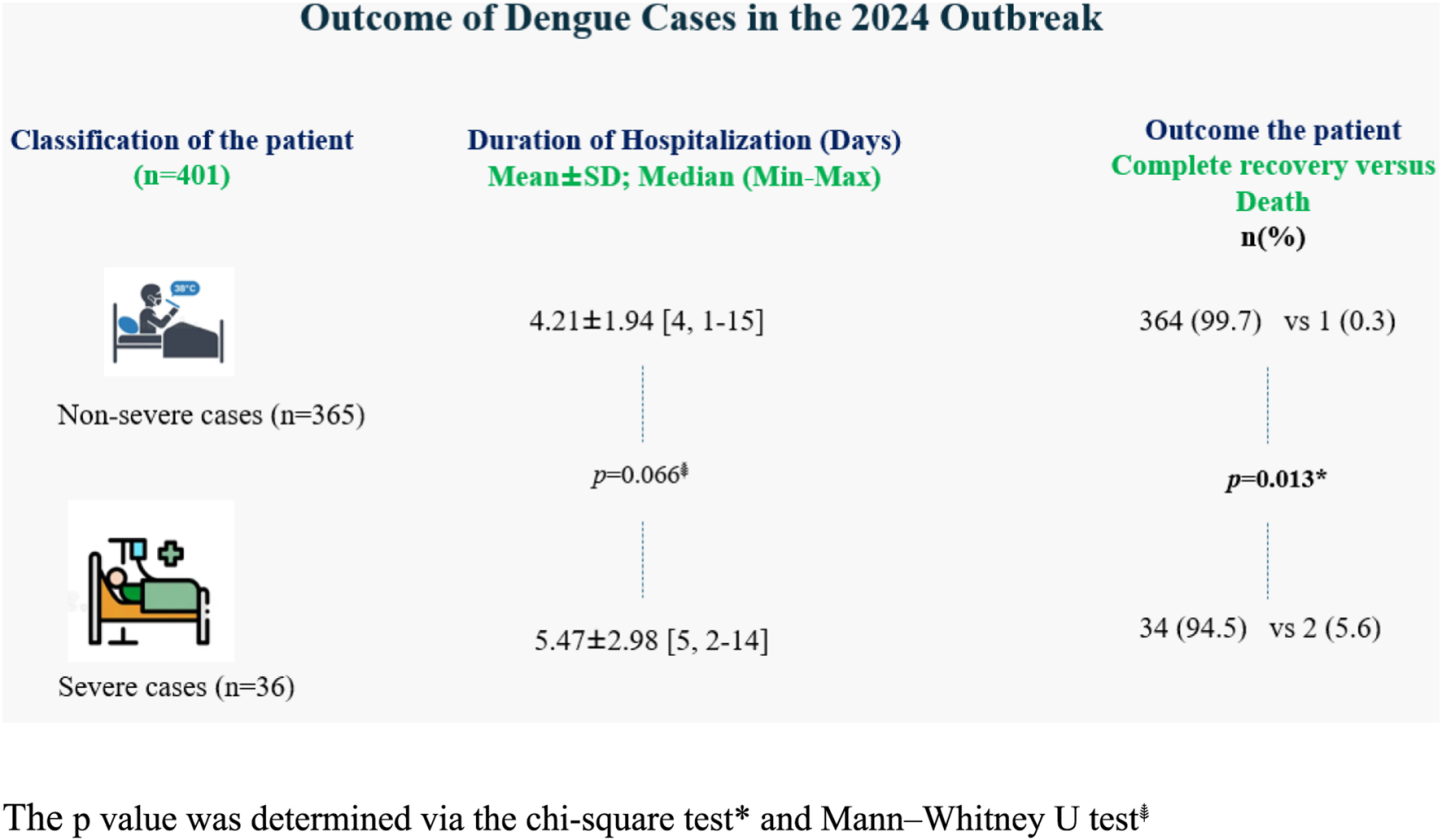
In-hospital outcomes according to the dengue severity classification (n = 401)

Overall, the in-hospital mortality rate among the study population was 0.75% (3 out of 401 patients). The median in-hospital stay was 4 days, ranging from 1 to 15 days (mean ± SD: 4.3 ± 2.05 days). Patients with severe dengue had significantly longer hospital stays than did those with nonsevere dengue. In-hospital mortality was significantly greater in patients with severe dengue fever than in those with nonsevere dengue (5.6% vs. 0.3%) (Fig. 2).

## Discussion

This study assessed the demographic characteristics and clinical presentations of patients with dengue fever during the 2024 outbreak in Bangladesh. This outbreak was associated with a relatively high prevalence of dengue in young adult males, with frequent gastrointestinal symptoms alongside general dengue manifestations. Almost one-tenth of the study patients had severe dengue, with a mortality of 0.75%.

The demographic profile of the dengue patients in our study revealed a predominance of young adults, with nearly half of the patients in the 2nd decade of life. This is consistent with a study conducted during the outbreak of 2023 in Bangladesh [8]. Recent studies conducted in Bangladesh and other regions of the world have reported a higher incidence of dengue among young adults [16, 23, 24]. Hence, the male predominance observed in the 2023 and 2024 studies is notably greater, with extreme gender disparities, and a deeper look at sociocultural or occupational factors that may increase exposure risk for males in the current outbreaks is needed. Almost three-fourths of the cases from urban areas align with the known epidemiology of dengue as a primarily urban disease due to the breeding preferences of Aedes mosquitoes [18]. However, the substantial number of rural cases suggests an expanding geographical distribution of dengue in Bangladesh, possibly indicating challenges in vector control in rural areas [17]. This study included 7.2% of the total sample of children aged 12–17 years, and all of these children were male.

The clinical profile of dengue patients in our study largely corresponds with established patterns, with fever being the most common presenting symptom. The high prevalence of headache (70.3%), myalgia (66.1%), and arthralgia (48.4%) is consistent with the classic symptomatology of dengue fever. However, a significant proportion of patients presented with gastrointestinal symptoms, particularly nausea (49.9%) and abdominal pain (43.9%), which aligns with the findings of recent outbreak studies [6, 7]. These findings emphasize the importance of considering dengue in the differential diagnosis of acute febrile illnesses with prominent gastrointestinal features. Respiratory symptoms, particularly cough (24.9%), were observed frequently in this study and may reflect an evolving clinical pattern in the current outbreak. This finding emphasizes the need for clinicians to maintain a high index of suspicion for dengue even in patients presenting with respiratory complaints, especially those with suspected coronavirus disease 2019 (COVID-19). Melena was the most common bleeding manifestation, reported in 14% patients. These findings are consistent with those of previous studies reporting a range of bleeding complications in patients with dengue fever [7, 25]. With respect to neurological presentations, this study revealed that dizziness was the most common symptom, reported in 15.7% of patients. Other neurological symptoms, such as vertigo, hallucinations, confusion, and loss of consciousness, have been reported in few patients. The presence of these neurological symptoms, even in a small proportion of patients, underscores the importance of careful neurological assessment in dengue patients.

Compared with adults, the adolescent dengue patients in this study presented significantly lower proportions of fever, headache and myalgia. Arthralgia and nausea were also less common in adolescents, whereas abdominal pain was more common. However, no statistical significance was observed. Research has suggested that adults with dengue fever frequently experience symptoms such as muscle pain, pain behind the eyes, nausea, and joint pain, whereas children are more likely to present with vomiting and skin rashes [26, 27]. In Bangladeshi children with dengue, common symptoms include fever and gastrointestinal issues [28]. However, the distinction between pediatric and adult symptoms remains vague [23]. The present study included patients aged 12 years or older, with only 7.2% being adolescents. No children aged less than 12 years were included in this study.

The median hemoglobin and hematocrit levels of the dengue patients were within normal ranges, as these data were collected on the day of admission when the disease had not yet progressed. The median white blood cell count was at the lower end of normal range, which is consistent with the leukopenia commonly observed in dengue fever patients. The mean platelet count was below normal, indicating thrombocytopenia, a hallmark of dengue. Elevated liver enzymes (ALT and AST) in many patients highlight hepatic involvement and the need to monitor liver function. No significant differences in hematological parameters or liver enzyme levels were found between children and adults.

The WHO severity classification revealed that a high proportion of patients with warning signs (Group B) emphasized the need for close monitoring and early intervention to prevent progression to severe dengue. The 9% prevalence of severe dengue (Group C) is concerning and highlights the potential for significant morbidity and mortality in the current outbreak. In Bangladesh, the 2023 dengue outbreak was particularly severe, with 17% of patients developing severe dengue, which is higher than that reported in the present study [29]. However, the present study focused only on the presentation on the day of admission, so disease severity was assessed before further progression of disease. Hence, 9% of severe cases on the day of admission are also considerably more common. In contrast with other regions, such as the Americas, where despite a record number of cases in 2023, only 0.16% of suspected cases were classified as severe [30]. Therefore, variability in outbreak severity exists across the world, which demands tailored public health responses according to the regional context. Additionally, factors such as the predominant circulating serotype and local healthcare infrastructure can significantly influence outcomes.

The clinical presentations of the study participants revealed significant differences between nonsevere and severe dengue cases. Notably, severe dengue patients presented increased frequencies of myalgia, fatigue, dizziness, vertigo, convulsions, abdominal distension, ascites, cough, breathlessness, shock, and features of plasma leakage. Hematocrit and platelet counts were also significantly greater in severe dengue patients than in nonsevere patients. Interestingly, the serum AST level was greater in nonsevere dengue patients than in nonsevere dengue patients, although this difference was not statistically significant. This could be because the data were collected on the day of admission. AST levels usually tend to rise during the febrile phase and may not necessarily correlate with disease severity at that point. Moreover, AST is released from multiple tissues, including skeletal muscle, cardiac muscle, and erythrocytes, and higher AST levels in nonsevere cases might reflect systemic inflammation or muscle damage.

Univariate logistic regression analysis revealed the following: fatigue/legargy; pale, cold, and clammy extremities; dizziness; features of plasma leakage; myalgia; dehydration; abdominal distension; vertigo; breathlessness; and cough and hematocrit. Multivariate regression analysis revealed that fatigue/lethargy (OR: 9.54; p < 0.001), pale, cold, clammy extremities (OR: 18.40; p < 0.001), features of plasma leakage (OR: 21.71; p < 0.001), and dizziness (OR: 2.55; p = 0.04) were independently associated with severe dengue. Therefore, critical symptoms are associated with severe dengue and should be monitored to ensure early identification and management [31].

The overall mortality rate in this study was 0.75%, which is lower than that reported in a previous study conducted in Bangladesh during the 2019 dengue outbreak [16]. Mortality was significantly greater among severe dengue patients, which is consistent with the findings of previous studies [32]. One patient initially admitted with nonsevere dengue later progressed to severe dengue and died. Therefore, the risk of clinical worsening exists even in cases initially classified as nonsevere. The median hospital stay was 4 days, ranging from a minimum of 1 day to a maximum of 15 days. The hospital stay was slightly longer in severe cases than in nonsevere cases, but the difference was not statistically significant.

This study has several limitations. Multiple follow-ups could not be done so, disease progression and course could not be captured. Additionally, investigations into virological and immunological factors contributing to disease severity could not be conducted. Future longitudinal research should be conducted to better understand these aspects of dengue infection.

## Conclusion

This study provides insights into the clinical and epidemiological profile of dengue patients during the 2024 outbreak in Bangladesh. A predominance of young adult males, a high prevalence of gastrointestinal symptoms alongside classic dengue manifestations, and a significant proportion of patients with warning signs and severe dengue were reported in this outbreak. These findings emphasize the need for early recognition and management of warning signs to prevent severe dengue. Future research should focus on longitudinal studies and investigations into factors contributing to disease severity.

## Data Availability

No datasets were generated or analysed during the current study.

## Abbreviations

CMCH: Chittagong Medical College Hospital
DMCH: Dhaka Medical College Hospital
SD: Standard deviation
ShSMCH: Shaheed Suhrawardy Medical College Hospital
